# Association Between Neutrophil-to-Lymphocyte Ratio and Early Detection of Ventilator-Associated Pneumonia in Mechanically Ventilated ICU Patients

**DOI:** 10.3390/jcm15103630

**Published:** 2026-05-09

**Authors:** Ioana Roxana Codru, Mihai Sava, Bogdan Ioan Vintila, Alina Simona Bereanu, Victoria Birlutiu

**Affiliations:** 1 Faculty of Medicine, Lucian Blaga University of Sibiu, 550024 Sibiu, Romania; ioanaroxana.bera@ulbsibiu.ro (I.R.C.); mihai.sava@ulbsibiu.ro (M.S.); victoria.birlutiu@ulbsibiu.ro (V.B.); 2 County Clinical Emergency Hospital of Sibiu, 550245 Sibiu, Romania

**Keywords:** NLR, VAP, ICU, mechanical ventilation, antibiotic stewardship

## Abstract

**Background**: Ventilator-associated pneumonia (VAP) is a frequent and severe complication in mechanically ventilated intensive care unit (ICU) patients. Early diagnosis remains challenging, and reliable biomarkers for early prediction are still needed. The neutrophil-to-lymphocyte ratio (NLR), derived from routine blood tests, has emerged as a potential marker of systemic inflammation. This study aimed to evaluate the association between NLR dynamics and the development of VAP in mechanically ventilated patients. **Methods**: This study represents a secondary analysis of a prospective observational cohort conducted in a tertiary ICU. Adult patients requiring mechanical ventilation for more than 48 h were included. Microbiological sampling and blood tests were performed at predefined time points. In the study group, endotracheal tubes were systematically replaced at scheduled intervals, while the control group received standard management. NLR values were calculated from routine complete blood counts and analyzed in relation to the occurrence of VAP. **Results**: A total of 312 patients were included (162 in the study group and 150 in the control group). VAP incidence was significantly lower in the study group (27.27%) compared with the control group (56.7%) (χ^2^ = 26.81, *p* = 2.24 × 10^−7^). Patients in the study group who developed VAP had higher NLR values prior to clinical diagnosis. ROC analysis identified an optimal NLR cutoff of 8.26 for predicting VAP. In contrast, NLR did not demonstrate predictive value in the control group (AUC = 0.55). NLR showed limited discriminatory ability (AUC ≈ 0.62), only slightly above random classification. NLR showed a moderate correlation with CRP (ρ = 0.46, *p* < 0.001) and a weaker association with PCT. **Conclusions**: Elevated NLR values were associated with the subsequent development of VAP; however, the observed predictive performance was modest and context-dependent. These findings should be considered exploratory and require validation in future studies.

## 1. Introduction

Ventilator-associated pneumonia (VAP) remains one of the most frequent and severe infections in mechanically ventilated patients admitted to intensive care units (ICUs) [1,2,3]. Despite advances in prevention strategies and antimicrobial therapy, VAP is still associated with increased mortality, prolonged duration of mechanical ventilation, extended ICU length of stay, and substantial healthcare costs [4]. Early diagnosis continues to be difficult due to nonspecific symptoms and the limitations of current diagnostic tools [5,6].

Traditional inflammatory biomarkers, such as C-reactive protein (CRP) and procalcitonin (PCT), are widely used in clinical practice to support the diagnosis of bacterial infections, including VAP. However, their diagnostic accuracy is limited by a lack of specificity in critically ill patients, and confounding factors such as trauma, surgery, or systemic inflammatory response syndrome. For this reason, there is growing interest in identifying readily available, cost-effective biomarkers that could improve early risk stratification and diagnostic precision in VAP [7,8].

The neutrophil-to-lymphocyte ratio (NLR) has emerged as a promising marker of systemic inflammation. Derived from routine complete blood count analysis, NLR reflects the balance between innate immune activation and adaptive immune suppression. Higher NLR values have been linked to poor outcomes in sepsis, community-acquired pneumonia, and various inflammatory and infectious diseases. In critically ill patients, NLR may indicate early immune dysregulation before clinical deterioration becomes evident [9,10].

Previous studies have reported AUC values for NLR ranging from approximately 0.72 to 0.95 in differentiating bacterial infections from non-infectious causes of inflammation. Additionally, NLR can aid in the early identification of bloodstream infections, particularly among elderly patients and those presenting with fever or sepsis [11,12,13,14]. NLR also has utility in predicting postoperative infectious complications, with pooled sensitivity and specificity around 0.77 and 0.78, respectively, and an AUC of 0.84 [15].

In viral infections, the predictive value of NLR is more variable. For example, NLR is less discriminative for influenza and RSV compared to COVID-19, where it is a stronger prognostic marker for poor outcomes (AUC 0.68 for COVID-19 vs. 0.57–0.58 for influenza/RSV) [16]. In tuberculosis, a higher NLR is associated with active disease, but cut-offs are inconsistent across studies [17]. In Mycoplasma pneumoniae pneumonia, NLR predicts poor outcomes such as necrotizing or refractory disease [18].

In the context of mechanical ventilation, sustained inflammatory stimulation, bacterial colonization, and biofilm formation on endotracheal tubes contribute to persistent immune activation [5,19]. These mechanisms may influence leukocyte dynamics and, consequently, NLR values. However, data regarding the role of NLR as an exploratory biomarker specifically associated with the development of VAP are limited and heterogeneous [20,21].

The aim of the present study was to evaluate the association between NLR dynamics and the occurrence of ventilator-associated pneumonia in mechanically ventilated ICU patients. We hypothesized that elevated NLR values precede the clinical diagnosis of VAP and provide complementary information to conventional inflammatory biomarkers, potentially supporting earlier identification of patients at increased risk.

## 2. Materials and Methods

### 2.1. Study Design and Setting

This study represents a secondary analysis of a prospective observational cohort conducted in the Intensive Care Unit of a tertiary county emergency hospital, following the protocol described by Codru et al. in 2023 in Medicina [6]. The original study was designed to evaluate the impact of preventive strategies targeting bacterial colonization and biofilm formation on endotracheal tubes in mechanically ventilated patients. Patient allocation to the study and control groups was determined by a predefined clinical protocol and was not randomized.

Microbiological Specimens—Timing of Collection, Sample Handling.

T0: Collection of tracheal aspirate within the first 2 h after patient admission to the intensive care unit or immediately after tracheal intubation and initiation of invasive respiratory support.

T1: Collection of a second tracheal aspirate at 48–72 h after T0. Replacement of the endotracheal tube at 48–72 h after T0, with collection of a tube specimen. The sample will be sonicated according to a standardized protocol to dislodge bacteria from the biofilm, and the resulting fluid will be inoculated into bacterial culture media.

T2: Collection of a third tracheal aspirate at 168–192 h after T0 in patients requiring prolonged mechanical ventilation. Replacement of the endotracheal tube at 168–192 h after T0, with collection of a new tube specimen, followed by sonication and inoculation of the resulting fluid onto culture media.

To ensure temporal coherence between microbiological dynamics and systemic inflammatory response, blood samples will be collected concomitantly with each respiratory specimen at T0, T1, and T2. This parallel sampling strategy enables longitudinal assessment of inflammatory biomarkers in relation to airway colonization and endotracheal tube biofilm development. Particular emphasis will be placed on the NLR, calculated from routine complete blood counts, as the primary variable of interest. By correlating NLR trends with microbiological findings at each predefined time point, the study aims to determine whether early NLR precedes and predicts the onset of VAP. This integrated approach allowed evaluating NLR not only as a static marker but also as a potential predictor of the most frequent respiratory complication in mechanically ventilated patients.

**Figure 1:**
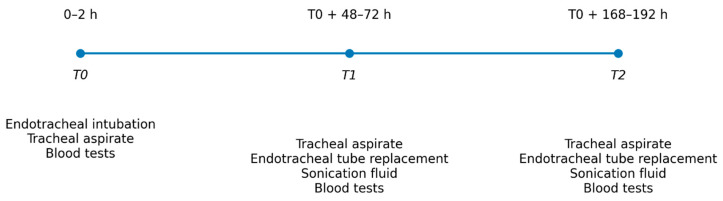
Graphical design of the study.

Within this framework, the present analysis focuses on the relationship between the host systemic inflammatory response and the microbiological dynamics occurring in the lower respiratory tract during mechanical ventilation. To enable this correlation, microbiological sampling was performed at predefined time points designed to capture the early phase of intubation, the intermediate period of airway colonization, and the later stage associated with biofilm maturation on the endotracheal tube. These standardized collection intervals provide a structured temporal context for evaluating both microbial colonization and the evolution of inflammatory markers, allowing subsequent integration with hematological parameters—particularly the NLR—investigated in this secondary analysis —as potential predictors of VAP (Figure 1).

### 2.2. Patient Population

**Figure 2:**
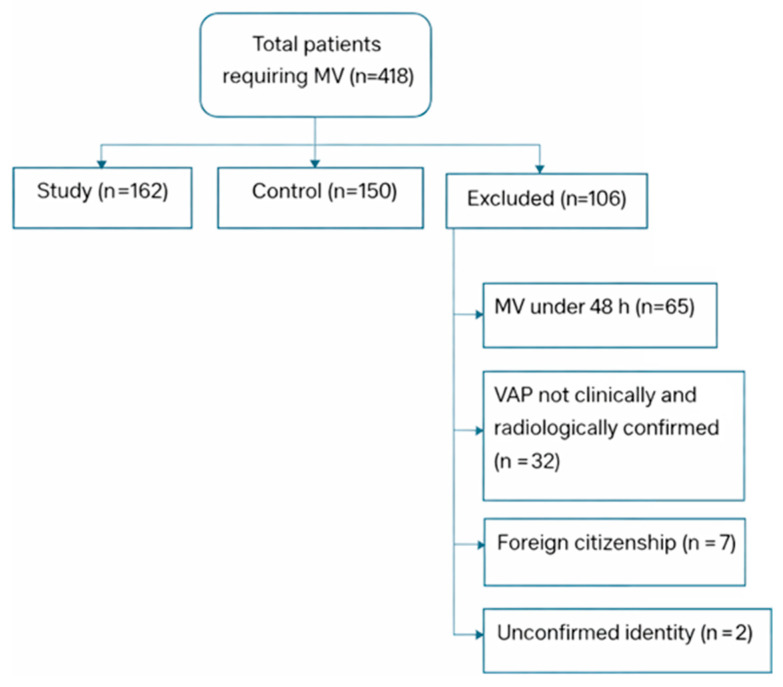
Study flow diagram of patient selection and group allocation.

A total of 418 patients requiring invasive mechanical ventilation were initially assessed for eligibility during the study period. Patients were excluded if they presented with pneumonia at the time of intubation, had documented immunosuppressive conditions (including hematologic malignancies or ongoing chemotherapy), or lacked complete clinical, laboratory, or microbiological data required for reliable classification of ventilator-associated pneumonia or calculation of the neutrophil-to-lymphocyte ratio. After applying these criteria, 312 patients were included in the final analysis, comprising 162 patients in the study group (scheduled endotracheal tube replacement) and 150 patients in the control group (standard management). A total of 106 patients were excluded, primarily due to incomplete clinical follow-up data, missing laboratory parameters necessary for NLR calculation, or insufficient microbiological data to establish VAP diagnosis according to predefined criteria. A complete-case analysis approach was applied, and no imputation methods were used due to the extent and heterogeneity of missing data. The study flow diagram detailing patient selection and exclusion is presented in Figure 2.

### 2.3. Definition of Ventilator-Associated Pneumonia

VAP was defined according to international guidelines as pneumonia occurring ≥48 h after initiation of invasive mechanical ventilation [22,23,24]. Characterized by new or progressive pulmonary infiltrates on chest imaging, at least two of the following: fever or hypothermia, leukocytosis or leukopenia, purulent respiratory secretions, microbiological confirmation from respiratory samples. Airway colonization was defined as the presence of microorganisms in respiratory samples without clinical or radiological evidence of infection, whereas VAP required both microbiological confirmation and compatible clinical and radiological criteria.

### 2.4. Data Collection

Demographic data, clinical characteristics, mechanical ventilation duration, and laboratory parameters were prospectively recorded. Complete blood count measurements were obtained at predefined time points during mechanical ventilation.

The neutrophil-to-lymphocyte ratio was calculated by dividing the absolute neutrophil count by the absolute lymphocyte count from the same blood sample. CRP and PCT levels were recorded when available and used for comparative analysis.

For correlation analyses of inflammatory biomarkers (NLR, CRP, and PCT), the entire study cohort was used to assess the general relationships among these parameters, independent of group allocation. In contrast, analyses evaluating the predictive performance of NLR for VAP development were conducted separately within the study and control groups, in order to account for differences in clinical management and microbiological monitoring. We adopted this approach to distinguish between general inflammatory associations and context-specific predictive performance.

### 2.5. Timing of NLR Assessment

NLR values were analyzed at predefined time points (T0, T1, T2). For predictive analysis, the NLR value at T0 was used as the primary variable, as it reflects the earliest available systemic inflammatory status before the development of ventilator-associated pneumonia.

Additional analyses were performed to evaluate the persistence of elevated NLR values over time (T1 and T2) in relation to the timing of clinical diagnosis. Notably, elevated NLR values were observed from the initial time point and remained persistently above the identified cutoff at subsequent time points, regardless of whether VAP was diagnosed early or later during mechanical ventilation.

### 2.6. Statistical Analysis

For statistical analysis, SPSS version 25.0 was used. This software enabled data processing and summarization using descriptive methods, including the calculation of measures of central tendency (mean, median) and dispersion (standard deviation, range) for quantitative variables, as well as the presentation of frequency distributions for qualitative variables. The use of SPSS facilitated a clear interpretation of the baseline characteristics of the studied population, providing a detailed overview of the collected data.

Subsequently, to compare differences between the study and control groups, appropriate inferential statistical tests were applied, adapted to the nature of the data. Depending on the distribution of the data, parametric tests such as the independent-samples t-test were used, or nonparametric tests such as the Mann–Whitney U test were used for variables that did not meet the assumption of normality. For categorical data, the Chi-square test was used, and multivariable logistic regression analyses were performed to identify predictive factors for VAP risk. Multivariable logistic regression models were constructed to evaluate the independent association between NLR and VAP, adjusting for available covariates, including age, sex, and duration of mechanical ventilation. The integration of these methods within SPSS enabled a robust evaluation of the data, contributing to the validity of the study’s conclusions.

This methodological approach allowed the evaluation of the direct impact of the applied protocol on the dynamics of bacterial colonization and its association with the development of ventilator-associated pneumonia, while also providing a solid basis for statistical comparisons between patients undergoing the intervention and those in the control group. Thus, the analysis aims not only to describe the clinical and microbiological characteristics of the studied population but also to establish relevant correlations between changes in the endotracheal biofilm and the risk of pulmonary infection. In this context, the neutrophil-to-lymphocyte ratio was analyzed as a potential predictive factor for VAP development in both the study and control groups, thereby assessing its prognostic value in relation to the dynamics of bacterial colonization and the clinical evolution of mechanically ventilated patients.

## 3. Results

### 3.1. Baseline Characteristics

A total of 418 patients requiring mechanical ventilation were initially assessed for eligibility. After applying the predefined inclusion and exclusion criteria, 312 patients were included in the final analysis, of whom 162 were allocated to the study group and 150 to the control group.

Among patients in the study group, 27.27% (*n* = 44) developed VAP, while 72.73% (*n* = 118) did not develop VAP during the observation period. In the control group, the incidence of ventilator-associated pneumonia was 56.7%, indicating a very high rate among mechanically ventilated patients.

The calculated Chi-square statistic was 26.81, and the obtained *p*-value was 2.24 × 10^−7^. Since this value is far below the conventional significance threshold of 0.05, the results indicate a statistically significant difference between the two groups, thereby rejecting the null hypothesis.

Baseline demographic and clinical characteristics were comparable between the VAP and non-VAP groups, with no statistically significant differences regarding age, sex distribution, or primary reason for ICU admission. The duration of mechanical ventilation was significantly longer in patients who developed VAP, regardless of group.

### 3.2. Neutrophil-to-Lymphocyte Ratio in VAP and Non-VAP Patients

Patients in the study group who received the preventive intervention targeting endotracheal tube biofilm and subsequently developed VAP had significantly higher NLR values than those who did not develop VAP. This difference was observed prior to the formal clinical diagnosis of VAP and persisted throughout the infectious episode. Median NLR values were consistently elevated in patients who developed VAP, reflecting a relative increase in neutrophil counts accompanied by lymphocyte depletion, whereas patients who remained free of VAP showed more stable NLR values during mechanical ventilation.

When analyzing the temporal dynamics of NLR, patients who developed VAP exhibited elevated NLR values from the earliest time point, prior to the clinical diagnosis of infection. These values remained consistently above the identified cutoff (≥8.26) at subsequent time points, regardless of the timing of VAP diagnosis, indicating a sustained inflammatory response. In contrast, patients who did not develop VAP showed lower and more stable NLR values across all time points.

To evaluate the predictive value of the NLR in relation to the development of VAP, a receiver operating characteristic (ROC) curve was generated. This analysis allows assessment of NLR’s ability to discriminate between patients who will develop VAP and those who will not. The resulting ROC curve showed a high area under the curve (AUC), indicating modest discriminatory performance for this biological marker.

**Figure 3:**
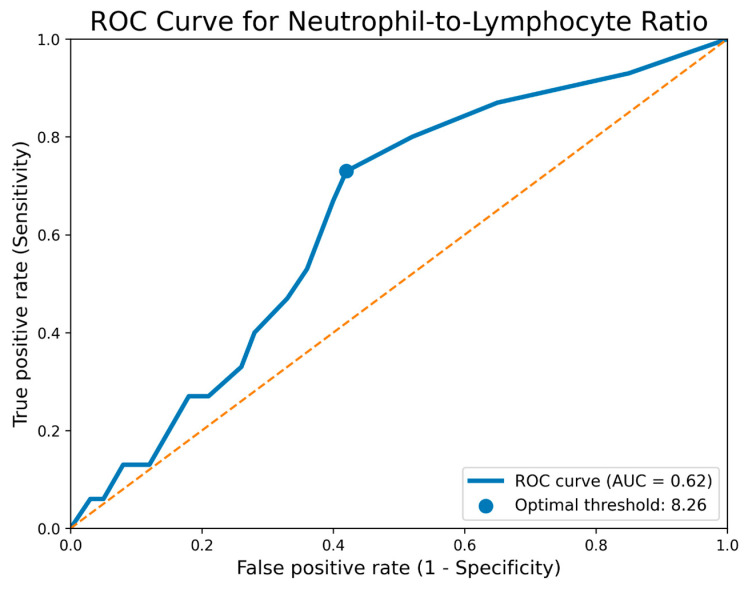
ROC Curve for Neutrophil-to-Lymphocyte Ratio in Predicting Ventilator-Associated Pneumonia.

As a reference for interpreting the results, the diagonal line of the ROC curve, corresponding to a random classification model (AUC = 0.5), was used to assess the superiority of NLR over a random distribution. The optimal NLR cutoff value for predicting VAP was determined using Youden’s Index, resulting in a value of 8.26. This threshold represents the best balance between sensitivity and specificity, suggesting that patients presenting with an NLR ≥ 8.26 at admission are more likely to develop VAP during hospitalization (Figure 3). The diagnostic performance analysis of the neutrophil-to-lymphocyte ratio (NLR) in predicting ventilator-associated pneumonia showed a sensitivity of 73.3% and a specificity of 57.9% at the optimal cutoff value of 8.26, established based on Youden’s Index.

In the control group, analysis of the NLR did not demonstrate predictive value for the development of VAP. The NLR values measured at admission showed distributions that were very similar between patients who subsequently developed VAP and those who did not. Specifically, the median NLR was 8.1 (IQR 5.9–10.7) in patients who developed VAP, compared with 7.8 (IQR 5.7–10.4) in patients who did not develop VAP; the difference was not statistically significant (*p* = 0.34). Similarly, the ROC analysis to assess the discriminatory ability of NLR between the two patient categories revealed an area under the curve of 0.55 (95% CI: 0.46–0.64), indicating low predictive performance, comparable to that of random classification. These findings suggest that, in the control group, NLR is not a useful predictive marker for VAP development, as the observed differences between subgroups were minimal and statistically nonsignificant.

### 3.3. Correlation with Conventional Inflammatory Biomarkers

Correlation analysis demonstrated a moderate positive association between NLR and C-reactive protein (CRP) values, indicating that increased NLR parallels systemic inflammatory activity (Spearman’s ρ = 0.46, *p* < 0.001). However, the strength of this correlation remained moderate, suggesting that NLR captures additional immunological information not fully reflected by CRP alone.

**Figure 4:**
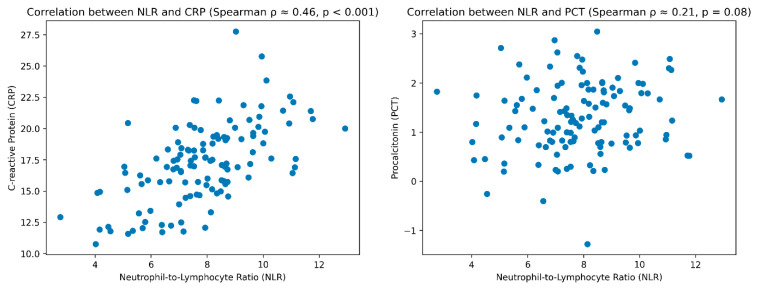
Correlation between traditional inflammatory biomarkers and NLR.

In contrast, the association between NLR and procalcitonin (PCT) was weaker and less consistent across time points (Spearman’s ρ = 0.21, *p* = 0.08). In several patients, elevations in NLR preceded measurable increases in PCT by approximately 24–48 h, highlighting a potential temporal advantage of NLR in the early identification of inflammatory deterioration (Figure 4).

## 4. Discussion

Ventilator-associated pneumonia remains one of the most frequent complications in mechanically ventilated patients and continues to represent a major challenge in intensive care medicine due to its impact on morbidity, mortality, and healthcare resource utilization. Early identification of patients at increased risk for VAP is particularly important because clinical and radiological signs often appear relatively late in the disease course. In this context, identifying simple, accessible biomarkers that signal early inflammatory changes could improve clinical decision-making and facilitate earlier diagnostic evaluation. The present study explored the role of the NLR as a potential early predictor of VAP in mechanically ventilated patients [18,25,26,27].

The present study evaluated the association between the neutrophil-to-lymphocyte ratio (NLR) and the development of ventilator-associated pneumonia (VAP) in mechanically ventilated ICU patients. The main finding is that elevated NLR values were associated with subsequent VAP development; however, the overall predictive performance was limited (AUC ≈ 0.62) and was not consistent across study groups.

These results suggest that NLR may reflect early systemic inflammatory changes preceding the clinical diagnosis of infection. However, the observed discriminatory ability was modest and only slightly above random classification, indicating limited standalone clinical utility.

The non-randomized design and protocol-based group allocation introduce substantial confounding. In particular, the scheduled endotracheal tube replacement applied in the study group represents a major intervention-related factor that may influence both VAP incidence and the apparent predictive performance of NLR. Therefore, it is difficult to distinguish the intrinsic predictive value of the biomarker from context-specific effects related to clinical management.

Notably, the predictive performance of NLR differed between the intervention and control groups. The absence of predictive value in the control group limits external validity and suggests that the association between NLR and VAP may be context-dependent rather than generalizable. The present study was not designed to investigate the mechanistic basis of these differences, and any causal interpretation would be speculative.

Compared with established biomarkers such as C-reactive protein (CRP) and procalcitonin (PCT), NLR demonstrated only moderate correlation and inconsistent association. Due to incomplete data availability, a direct head-to-head comparison of diagnostic performance was not feasible, and NLR should therefore be interpreted as a complementary rather than a substitutive marker.

From a clinical perspective, the main advantage of NLR lies in its availability and low cost, as it is derived from routine blood tests. However, given its limited discriminatory performance and lack of external validation, NLR cannot be recommended as a standalone predictor for VAP.

These findings are consistent with previous studies demonstrating that NLR reflects the balance between innate immune activation and adaptive immune suppression during systemic inflammation. Neutrophilia is typically associated with acute bacterial infection and inflammatory signaling, whereas lymphopenia may result from stress-induced immune dysregulation and lymphocyte apoptosis during critical illness. The resulting increase in NLR therefore represents a composite marker of immune activation and immunosuppression, which may explain its usefulness as an early indicator of infectious complications in critically ill patients [11,12,13,14,15,16,17,18,28].

The observed difference in predictive performance between the study and control groups remains difficult to interpret. The absence of predictive value in the control group limits external validity and suggests that the association between NLR and VAP may be context-dependent rather than generalizable. Importantly, this inconsistency represents the study’s most significant limitation, as it substantially limits the generalizability of the findings.

The observed difference in VAP incidence between groups is likely driven by the preventive intervention rather than differences in inflammatory biomarkers. Therefore, the independent predictive value of NLR should be interpreted cautiously.

Formal interaction analysis between group allocation and NLR was not performed due to limited statistical power.

However, the present study did not include a detailed analysis of microbiological profiles, airway colonization dynamics, or potential direct effects of the intervention on systemic inflammatory parameters. Therefore, the observed differences between groups should be considered hypothesis-generating. Further studies integrating microbiological, immunological, and clinical data are needed to clarify whether and how preventive strategies targeting biofilm formation can improve the predictive performance of inflammatory biomarkers, such as NLR.

The study included a preventive protocol involving scheduled endotracheal tube replacement, which may have influenced microbiological dynamics and inflammatory responses. However, the present analysis focuses on the relationship between NLR and VAP development, and the impact of the intervention itself was not the primary objective.

However, several limitations should be acknowledged. First, the study was conducted in a single center, which may limit the generalizability of the findings to other ICU settings with different patient populations or clinical practices. Multicenter studies including diverse patient populations are required to validate these results. Second, although the study was based on a prospective cohort, the present analysis represents a secondary evaluation of the collected data, and therefore, residual confounding factors cannot be entirely excluded. Third, inflammatory biomarkers may be influenced by multiple factors common in critically ill patients, including trauma, surgery, systemic inflammatory response syndrome, or concomitant infections. Additionally, detailed information on comorbidities and underlying clinical conditions was not consistently available, which may have influenced NLR values. Therefore, the potential impact of patient-specific factors on the observed associations cannot be fully excluded. Another limitation of this study is the lack of detailed information regarding the primary indications for mechanical ventilation and certain clinical outcomes, such as ICU mortality and length of stay. As this analysis was based on a secondary dataset, these variables were not consistently available for all patients. Consequently, the applicability of the findings to specific ICU subpopulations cannot be fully assessed. Future studies incorporating comprehensive clinical characterization are warranted to better define the role of NLR across different categories of critically ill patients. Residual confounding cannot be excluded due to the non-randomized design and incomplete availability of severity indices and comorbidity data. Finally, although the ROC analysis demonstrated a promising threshold for NLR, this cut-off value should be interpreted cautiously and validated in larger multicenter studies. Detailed quantitative analysis of NLR dynamics across all time points was limited by data availability, and future studies should provide comprehensive longitudinal assessments.

Key clinical outcomes, including ICU mortality, length of ICU stay, and duration of mechanical ventilation, were not consistently available and therefore could not be analyzed.

Future research should aim to validate NLR’s predictive performance in larger cohorts and to explore its integration with other clinical and microbiological parameters in predictive models for VAP. A detailed analysis of pathogen-specific differences in NLR response was not performed in the present study. As different microorganisms may induce distinct inflammatory patterns, future studies should explore whether NLR dynamics vary according to the causative pathogen. Combining NLR dynamics with microbiological surveillance strategies, such as biofilm analysis and molecular pathogen detection, may provide a more comprehensive approach to early infection detection in mechanically ventilated patients. Also, pathogen-specific analyses and evaluation of the relationship between microbial etiology and NLR were not performed and represent an important area for future research.

In the context of antimicrobial stewardship (AMS), NLR, along with other biomarkers or scores, may help identify patients who may benefit from prompt antibiotic initiation or de-escalation, thereby supporting AMS principles of optimizing diagnosis, drug selection, and therapy duration. AMS programs, as described in the medical literature, emphasize the importance of multidisciplinary teams and individualized approaches, especially in high-risk populations such as those with neutropenia, where early and appropriate antibiotic choice is critical [29].

Overall, NLR appears to reflect systemic inflammatory response rather than serving as a reliable standalone predictor of VAP, and its apparent utility may depend on clinical context and intervention-related factors.

## 5. Conclusions

The findings of this study suggest that NLR may be associated with inflammatory changes preceding the development of VAP in mechanically ventilated ICU patients. However, its predictive performance was modest and limited to a specific clinical context, and no direct comparison with established biomarkers was possible. These results do not support the use of NLR as a robust or independent predictor of VAP. Rather, they highlight the potential context-dependent behavior of inflammatory biomarkers and should be considered exploratory and hypothesis-generating, requiring validation in future studies.

The predictive performance of the NLR was enhanced in the patient group that employed microbiological monitoring and preventive strategies to manage endotracheal tube colonization. This finding indicates that combining systemic inflammatory markers with microbiological surveillance could improve early detection of VAP risk.

Due to its simplicity and availability, NLR may serve as a practical adjunct for early risk stratification and may provide complementary information, but it is not sufficient for clinical decision-making when stewardship is the target.

## Data Availability

Data are contained within this article.

## References

[1] Hlinkova S., Moraucikova E., Strzelecka A., Mrazova M., Littva V. (2025). Ventilator-Associated Pneumonia in Intensive Care Units: A Comparison of Pre-Pandemic and COVID-19 Periods. J. Clin. Med..

[2] Howroyd F., Gill R., Thompson J., Smith F.G., Nasa P., Gopal S., Duggal N.A., Ahmed Z., Veenith T. (2025). Ventilator-Associated Pneumonia: Mechanisms, an Appraisal of Current Therapies and the Role for Inhaled Antibiotics in Prevention and Treatment. Respir. Med..

[3] Sadigov A., Mamedova I., Mammmadov K. (2019). Ventilator-Associated Pneumonia and In-Hospital Mortality: Which Risk Factors May Predict In-Hospital Mortality in Such Patients?. J. Lung Health Dis..

[4] Ochoa P., Mendoza A.R., Molano D., Masclans J.R., Parada-Gereda H.M. (2025). Risk Factors and Outcomes of Ventilator-Associated Pneumonia: An Updated Systematic Review and Meta-Analysis. BMC Pulm. Med..

[5] Codru I.R., Vintilă B.I., Sava M., Bereanu A.S., Neamțu S.I., Bădilă R.M., Bîrluțiu V. (2024). Optimizing Diagnosis and Management of Ventilator-Associated Pneumonia: A Systematic Evaluation of Biofilm Detection Methods and Bacterial Colonization on Endotracheal Tubes. Microorganisms.

[6] Codru I.R., Sava M., Vintilă B.I., Bereanu A.S., Bîrluțiu V. (2023). A Study on the Contributions of Sonication to the Identification of Bacteria Associated with Intubation Cannula Biofilm and the Risk of Ventilator-Associated Pneumonia. Medicina.

[7] Sdougka M., Simitsopoulou M., Volakli E., Violaki A., Georgopoulou V., Ftergioti A., Roilides E., Iosifidis E. (2023). Evaluation of Five Host Inflammatory Biomarkers in Early Diagnosis of Ventilator-Associated Pneumonia in Critically Ill Children: A Prospective Single Center Cohort Study. Antibiotics.

[8] Póvoa P., Coelho L., Cidade J.P., Ceccato A., Morris A.C., Salluh J., Nobre V., Nseir S., Martin-Loeches I., Lisboa T. (2024). Biomarkers in Pulmonary Infections: A Clinical Approach. Ann. Intensive Care.

[9] Tekin A., Wireko F.W., Gajic O., Odeyemi Y.E. (2024). The Neutrophil/Lymphocyte Ratio and Outcomes in Hospitalized Patients with Community-Acquired Pneumonia: A Retrospective Cohort Study. Biomedicines.

[10] Høiby N., Bjarnsholt T., Moser C., Bassi G.L., Coenye T., Donelli G., Hall-Stoodley L., Holá V., Imbert C., Kirketerp-Møller K. (2015). ESCMID∗ Guideline for the Diagnosis and Treatment of Biofilm Infections 2014. Clin. Microbiol. Infect..

[11] Chen Y., Ye L., Wu Y., Shen B., Zhang F., Qu Q., Qu J. (2020). Neutrophil-Lymphocyte Ratio in Predicting Infective Endocarditis: A Case-Control Retrospective Study. Mediat. Inflamm..

[12] Li P., Zhang J., Chen K., Pei Q., Ding M., Wang C. (2025). Neutrophil-to-Lymphocyte Ratio: A Potential Supportive Marker for Elderly Community-Acquired Bloodstream Infections—A Retrospective Study. PeerJ.

[13] Anoun J., Ajmi M., Riahi S., Dhaha Y., Mbarki D., ben Hassine I., Romdhane W., Baya W., Adaily N., Mzabi A. (2024). Neutrophil-to-Lymphocyte and Platelet-to-Lymphocyte Ratios in Bacterial Infections: Contributions to Diagnostic Strategies in a Tertiary Care Hospital in Tunisia. F1000Research.

[14] Russell C.D., Parajuli A., Gale H.J., Bulteel N.S., Schuetz P., de Jager C.P.C., Loonen A.J.M., Merekoulias G.I., Baillie J.K. (2019). The Utility of Peripheral Blood Leucocyte Ratios as Biomarkers in Infectious Diseases: A Systematic Review and Meta-Analysis. J. Infect..

[15] Qian B., Zheng Y., Jia H., Zheng X., Gao R., Li W. (2023). Neutrophil-Lymphocyte Ratio as a Predictive Marker for Postoperative Infectious Complications: A Systematic Review and Meta-Analysis. Heliyon.

[16] Prozan L., Shusterman E., Ablin J., Mitelpunkt A., Weiss-Meilik A., Adler A., Choshen G., Kehat O. (2021). Prognostic Value of Neutrophil-to-Lymphocyte Ratio in COVID-19 Compared with Influenza and Respiratory Syncytial Virus Infection. Sci. Rep..

[17] Fritschi N., Vaezipour N., Buettcher M., Portevin D., Naranbhai V., Ritz N. (2023). Ratios from Full Blood Count as Markers for TB Diagnosis, Treatment, Prognosis: A Systematic Review. Int. J. Tuberc. Lung Dis..

[18] Li D., Gu H., Chen L., Wu R., Jiang Y., Huang X., Zhao D., Liu F. (2023). Neutrophil-to-Lymphocyte Ratio as a Predictor of Poor Outcomes of Mycoplasma Pneumoniae Pneumonia. Front. Immunol..

[19] Codru I.R., Vintilă B.I., Bereanu A.S., Sava M., Popa L.M., Birlutiu V. (2025). Antimicrobial Resistance Patterns and Biofilm Analysis via Sonication in Intensive Care Unit Patients at a County Emergency Hospital in Romania. Pharmaceuticals.

[20] Silva N.B.S., Marques L.A., Röder D.D.B. (2021). Diagnosis of Biofilm Infections: Current Methods Used, Challenges and Perspectives for the Future. J. Appl. Microbiol..

[21] Flurin L., Greenwood-Quaintance K.E., Esper R.N., Sanchez-Sotelo J., Patel R. (2021). Sonication Improves Microbiologic Diagnosis of Periprosthetic Elbow Infection. J. Shoulder Elb. Surg..

[22] Klompas M., Branson R., Cawcutt K., Crist M., Eichenwald E.C., Greene L.R., Lee G., Maragakis L.L., Powell K., Priebe G.P. (2022). Strategies to Prevent Ventilator-Associated Pneumonia, Ventilator-Associated Events, and Nonventilator Hospital-Acquired Pneumonia in Acute-Care Hospitals: 2022 Update. Infect. Control Hosp. Epidemiol..

[23] Chen J., Yasrebinia S., Ghaedi A., Khanzadeh M., Quintin S., Dagra A., Peart R., Lucke-Wold B., Khanzadeh S. (2023). Meta-Analysis of the Role of Neutrophil to Lymphocyte Ratio in Neonatal Sepsis. BMC Infect. Dis..

[24] Alves J., Abreu B., Palma P., Alp E., Vieceli T., Rello J. (2023). Antimicrobial Stewardship on Patients with Neutropenia: A Narrative Review Commissioned by Microorganisms. Microorganisms.

[25] Ncezid (2026). DHQP Pneumonia (Ventilator-Associated [VAP] and Non-Ventilator-Associated Pneumonia [PNEU]) Event. https://www.cdc.gov/nhsn/psc/pneu/index.html.

[26] Howroyd F., Chacko C., MacDuff A., Gautam N., Pouchet B., Tunnicliffe B., Weblin J., Gao-Smith F., Ahmed Z., Duggal N.A. (2024). Ventilator-Associated Pneumonia: Pathobiological Heterogeneity and Diagnostic Challenges. Nat. Commun..

[27] Kalanuria A.A., Zai W., Mirski M. (2014). Ventilator-Associated Pneumonia in the ICU. Crit. Care.

[28] Guillamet C.V., Kollef M.H. (2024). Is Zero Ventilator-Associated Pneumonia Achievable? Updated Practical Approaches to Ventilator-Associated Pneumonia Prevention. Infect. Dis. Clin. N. Am..

[29] Colaneri M., Montrucchio G., Scaglione G., Monti G., Tricella G., Genovese C., Agostini F., Dore F., Viaggi B., Brazzi L. (2025). Incidence, Microbiology, and Mortality of Ventilation-Associated Pneumonia in a Large Italian Cohort of Critically Ill Patients: Results from the PROSAFE Project. Clin. Microbiol. Infect..

